# Anemia and Iron Deficiency in Cardiac Surgery Patients: Prevalence, Implications, and Therapeutic Considerations

**DOI:** 10.3390/jcm14228261

**Published:** 2025-11-20

**Authors:** Isabelle Frei, Annika Léonie Gogniat, Andreas Buser, Daniel Bolliger

**Affiliations:** 1 Clinic for Anaesthesia, Intermediate Care, Prehospital Emergency Medicine and Pain Therapy, University Hospital Basel, University of Basel, 4031 Basel, Switzerland; isabelle.frei@usb.ch (I.F.); annika.gogniat@usb.ch (A.L.G.); 2 Medical Faculty, University of Basel, 4056 Basel, Switzerland; andreas.buser@usb.ch; 3 Regional Blood Transfusion Service, Swiss Red Cross, 4031 Basel, Switzerland; 4 Clinic of Hematology, University Hospital Basel, University of Basel, 4031 Basel, Switzerland

**Keywords:** anemia, patient blood management, transfusion, cardiac surgery, preoperative optimization

## Abstract

Preoperative anemia and non-anemic iron deficiency are common in cardiac surgery patients. Both are frequently caused by absolute iron deficiency or are associated with chronic diseases and inflammation. Multiple studies have shown an association between preoperative anemia and increased RBC transfusion, prolonged intensive care unit and hospital length of stay, and higher postoperative morbidity and mortality. The impact of preoperative non-anemic iron deficiency on postoperative adverse outcome is less clear, but worsened postoperative outcomes have been suggested. Accordingly, treatment for anemia and iron deficiency is a primary goal in preoperative optimization in cardiac surgery patients. Several guidelines recommend timely supplementation with modern oral iron formulation as first-line intervention, followed by intravenous iron administration in case of patient intolerance or time restriction. In patients with non-pure iron deficiency or in ultra-short treatment strategies, the combined therapy of intravenous iron and erythropoiesis-stimulating agents seems safe and most efficacious to increase red blood mass before surgery. However, the positive effects of preoperative interventions in anemic or iron-deficient patients were mostly limited to reduced transfusion of red blood cells, whereas lower morbidity and mortality were shown in limited studies only. It is also important to note that allogeneic blood products are a limited resource, and preoperative oral iron supplementation showed the best cost-effectiveness.

## 1. Introduction

Anemia is a common comorbidity in the general population, with an estimated global prevalence of about 30%. In the elderly, the prevalence of anemia is even higher [1]. Anemia is most frequently caused by iron deficiency followed by anemia associated with chronic diseases. Of note, the prevalence and common pathomechanisms of anemia might differ with age, sex, ethnicities, and geographic locations [1,2].

In patients presenting for cardiac surgery, anemia has been reported with similar and even higher prevalence than in the general population [3,4,5]. Preoperative iron deficiency, combined with or without anemia, seems to be even more common with a reported incidence between 30% and 80% [4,6]. Lower hemoglobin values are associated with the higher requirements for perioperative transfusion of multiple blood products and, most importantly, with higher perioperative mortality [7,8]. The need for perioperative blood transfusion might additionally worsen patient outcome, increase morbidity and mortality, and prolong intensive care unit (ICU) and hospital length of stay (LOS) [4].

The association between perioperative allogeneic blood usage and increased morbidity and mortality has recently gained significant attention, especially in patients undergoing cardiac surgery [9,10]. In order to reduce perioperative blood transfusion and, ultimately, improve patient outcomes, the Word Health Organization (WHO) and multiple medical societies have promoted and endorsed the implementation of patient blood management (PBM) programs [11]. In the perioperative setting, PBM programs consist of a bundle of interventions focusing on the pre-, intra-, and postoperative periods, which have been extensively described [11,12,13,14,15,16].

**Figure 1:**
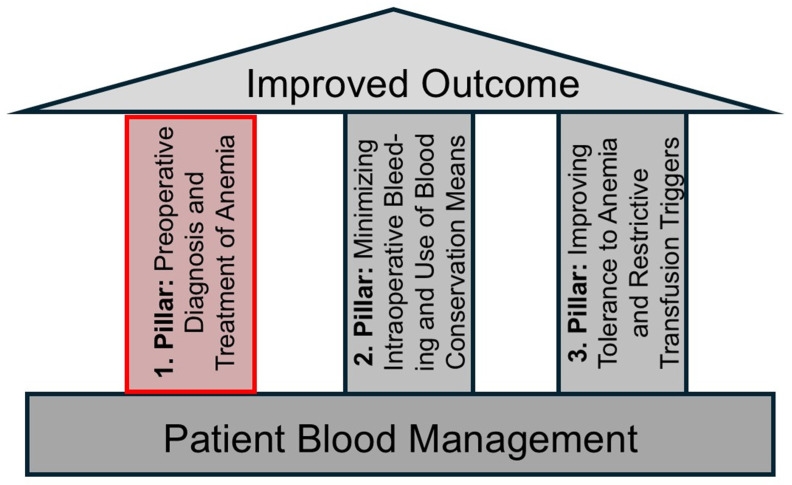
The three pillars of perioperative patient blood management (PBM). This review focuses on the first pillar.

This narrative review focuses on the first pillar of PBM in cardiac surgery (Figure 1), i.e., the preoperative diagnosis and treatment of anemia to boost the patient’s own blood reserves. In addition, we summarize the recent evidence of preoperative interventions in anemic or iron-deficient patients and their potential benefits for patient outcome. Finally, we discuss the cost-effectiveness of such preoperative interventions.

## 2. Search Strategies

An extensive literature search in PubMed was performed using the following terms: (preoperative) AND (anemia) AND (cardiac surgery) on 1 September 2025. The search identified 951 publications. The authors excluded publications that were in languages other than English and pediatric cardiac surgery studies, focusing on clinical studies, clinical trials, meta-analyses, randomized controlled trials (RCT), and systematic and non-systematic reviews published in the last 10 years. After reading the abstracts, publications investigating the potential impact of preoperative anemia on outcomes and interventions on anemic or iron-deficient patients before cardiac surgery were critically assessed and eventually included in this review.

## 3. Preoperative Anemia in Cardiac Surgery Patients

### 3.1. Definition of Anemia

According to the WHO definition from 1967, anemia is diagnosed in patients with hemoglobin levels <120 g/L in women and <130 g/L in men. Further, anemia is subclassified in adult men and non-pregnant women as mild (Hb 110–120/130 g/L), moderate (80–109 g/L), and severe (<80 g/L). Although the WHO definitions of anemia are frequently used in studies and research, one must consider that “normal” hemoglobin levels vary with age, physiognomics, or ethnicity [13,17,18,19]. Accordingly, the WHO thresholds have recently been questioned [20], and the 2017 European consensus statement suggested to use a pragmatic threshold of 130 g/L for all preoperative patients [21]. Further, hemoglobin or hematocrit values below suggested thresholds do not necessarily predict the increased requirements for red blood cell (RBC) transfusion during cardiac surgery [18,22]. It has been suggested to calculate the total RBC mass (i.e., total blood volume x hematocrit) to estimate the risk of perioperative RBC transfusion, as it might be better suited to initiate therapeutic interventions than hematocrit or hemoglobin values [13,22]. For example, the total RBC mass in a very small person with low-normal hemoglobin values might not be high enough to avoid RBC transfusion in extensive cardiac surgery, whereas patients with larger body weight might be less likely to be transfused even with hemoglobin levels <120 g/L. It remains, however, unclear whether total RBC mass shows a better association with postoperative morbidity and mortality than hemoglobin values.

Finally, the WHO thresholds of <120 g/L in woman and <130 g/L in men have also been applied to diagnose anemia in the intra- and postoperative setting [23]. However, this might be questionable as up to 90% of patients fulfill the criterion of at least mild anemia after cardiac surgery [23]. Further, RBC transfusion is only indicated in patients with severe anemia (i.e., hemoglobin values <75 g/L) or signs of inadequate tissue oxygenation [13].

### 3.2. Definition of Iron Deficiency

Although a variety of definitions are used incorporating different cutoffs of multiple laboratory parameters, perioperative iron deficiency is commonly defined as either a ferritin level <100 μg/L or a ferritin level <300 μg/L in combination with transferrin saturation <20% [14,24,25]. Of note, normal ferritin levels are age-dependent, and especially in younger patients the lower thresholds of ferritin (20–30 μg/L) are used to define iron deficiency [26].

### 3.3. Common Types of Anemia

The most common cause of anemia worldwide is iron deficiency—either absolute due to blood loss or reduced nutritional intake, or functional due to iron sequestration. The latter can be found in patients with chronic inflammation or insufficient levels of erythropoiesis-stimulation agents (Table 1) [27]. Alternative terms of functional iron deficiency anemia are anemia of chronic disease, anemia of inflammation, or non-pure iron deficiency anemia. The respective term is usually chosen based on the pathomechanism of anemia. Finally, anemia is unexplained in up to 30% of elderly patients. This so-called unexplained anemia of the elderly (UAE) is a complex entity with respect to diagnosis, pathomechanism, and therapy.

### 3.4. Iron Deficiency Anemia

Approximately 30% to 50% of anemic patients presenting for cardiac surgery have absolute iron deficiency with depleted iron stores, mainly indicated by low serum ferritin (<100 ng/mL), low transferrin saturation (<20%), and low reticulocyte count due to different pathomechanisms (Table 1) [4,15,28]. In agreement, a recent Cochrane review identified iron deficiency as the leading cause of anemia in different surgical populations including cardiac surgery [29]. However, most larger studies and meta-analyses often did not exactly differentiate between absolute and functional iron deficiency.

### 3.5. Anemia of Chronic Disease

Anemia of chronic disease or anemia of inflammation is also frequently observed in cardiac surgery patients. Although it might be associated with absolute iron deficiency, anemia of chronic disease is primarily characterized by functional iron deficiency. Accordingly, ferritin levels are sufficient or elevated due to chronic inflammation, whereas transferrin saturation remains low, reflecting limited circulating iron availability for erythropoiesis. Morphologically, this type of anemia often presents as normocytic and normochromic (Table 2). The pathomechanism of anemia of chronic disease is primarily a disturbance of iron hemostasis, which is mainly immune-drive and associated with dysregulation of cytokines. Up- or downregulated inflammatory cytokines inhibit iron absorption via increased expression of hepcidin from the liver resulting in iron dysregulation [30]. Iron storage is increased in macrocytes; however, this iron is poorly available for erythropoiesis. Finally, the expression of erythropoietin-receptors is down-regulated, resulting in reduced stimulation of erythropoiesis [30,31].

Functional iron deficiency might account for about 30–50% of anemia cases in cardiac surgery patients [15]. In a large cohort of more than 10,000 elective cardiac surgery patients, 87% of anemic patients had normocytic anemia, potentially consistent with anemia of chronic disease [32]. The predominant pattern of normocytic anemia in cardiac surgery patients might reflect the high prevalence of relevant co-morbidities including reduced renal function and chronic low-grade inflammation, such as in severe atherosclerosis. In agreement, a recent Cochrane review identified anemia of chronic disease as a major contributor to preoperative anemia in surgical patients, particularly in those with increased cardiovascular comorbidities [29]. Recently, therapeutic iron administration in patients with heart failure has gained interest. Of note, heart failure itself might cause inflammation and thereby functional iron deficiency [24,33].

Finally, anemia associated with chronic kidney disease shares some characteristics with anemia of chronic disease [31]. However, decreased erythropoietin production mediated by renal insufficiency might contribute more importantly than in other forms of anemia of chronic disease.

### 3.6. Laboratory Testing in Anemia

Diagnosis of anemia is simple. However, the complex and multifactorial biology of anemia (Table 1) requires extended laboratory testing and expert knowledge to initiate adequate therapy. Varying screening algorithms have been described to differentiate between the different types of anemia [15,31]. Generally, it is recommended to perform a full blood count including reticulocyte count, ferritin levels, transferrin concentration, transferrin saturation, and levels of soluble transferrin receptors (Table 2) [15]. The hemoglobin content of the reticulocytes (CHr) might have additional diagnostic value, as it specifies iron availability for erythropoiesis during the last 5 days. Further, C-reactive protein (CRP) and creatinine levels should be evaluated in anemic patients for more comprehensive therapeutic decision-making. Some laboratories offer the possibility to test for erythropoietin levels. However, absolute erythropoietin levels are often difficult to interpret and are of limited value, especially in patients with anemia of chronic disease [15,34]. Finally, more elaborate laboratory tests might be necessary in patients with specific hematologic diseases (e.g., myelodysplastic syndromes) or inborn hemoglobinopathies. In such patients, advice from hematologists seems imperative to optimize preoperative therapy [15].

### 3.7. Specific Consideration Before Cardiac Surgery

Timely diagnosis and therapeutic interventions, ideally performed by the patient’s general practitioner, should be performed in anemic patients before cardiac surgery [15,35]. In most clinical settings, however, diagnosis and therapeutic interventions are carried out by surgeons and/or anesthesiologists. Simplified algorithms allowing hematology-naïve health professionals to differentiate between common types of anemia might be optimal in combination with easy-to-perform interventions [14,15]. Of importance, no optimal algorithm for the different institutions is available and the individualized algorithms adopted the specific local conditions might be recommended.

**Figure 2:**
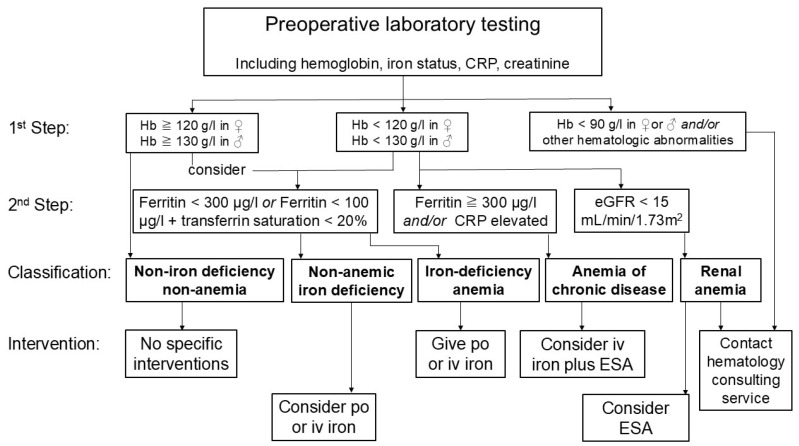
Simplified diagnostic algorithm and interventions as part of patient blood management in cardiac surgery. Based on few laboratory tests including hemoglobin level, ferritin levels, transferrin saturation, C-reactive protein (CRP) and estimated glomerular filtration (eGFR) rate calculated by creatinine level, patients can be divided into following groups: Non-iron-deficient non-anemic, iron-deficient non-anemic, iron-deficiency anemia, anemia of chronic disease, and renal anemia. Based on this simplified diagnostic algorithm, non-specialist physicians can decide for optimal therapy including peroral (po) or intravenous (iv) iron substitution or administration of erythropoiesis-stimulating agents (ESA).

It has been suggested that patients can usually be assigned to groups of iron deficiency without anemia, iron deficiency anemia, renal anemia, anemia of inflammation, and non-iron-deficient non-anemia based on limited laboratory testing including blood count, ferritin levels, CRP, and creatinine (Figure 2) [15]. This simplified screening would allow for easy and timely treatment decisions that can be enacted by a non-hematology specialist. Of note, therapeutic interventions including iron transfusion should not be generally used in all patients, but should be individualized based on laboratory testing [14,36].

## 4. Impact of Anemia on Outcome After Cardiac Surgery

### 4.1. Association of Preoperative Anemia and Adverse Outcomes

Multiple studies in patients undergoing cardiac surgery showed an association between preoperative anemia using the WHO definitions and increased intraoperative RBC transfusion, postoperative mortality and morbidity including myocardial ischemia/infarction, acute kidney injury (AKI), neurological complications, infections, and prolonged ICU and hospital LOS (Table 3) [3,4,37,38]. In agreement, a recent systematic review and meta-analysis including 42 studies with >160,000 patients reported that anemia was associated with a 2.5-fold increase in mortality (OR 2.52, 95% CI 2.21–2.87, *p* < 0.001) [39]. The increasing severity of anemia was associated with higher risk of mortality, meaning that anemic patients with lower hemoglobin have a higher mortality risk than with the higher hemoglobin value [37,39]. Requirements for RBC transfusion were about 4-fold higher (OR 4.0, 95% CI 3.4–4.7) in anemic patients compared to non-anemic patients [39]. Finally, the risk of postoperative infections, stroke, AKI, cardiac ischemia, and pulmonary complications were about 1.5–2.5-fold higher in anemic patients compared to non-anemic patients [39]. Accordingly, anemia was associated with prolonged ICU and hospital LOS. Of note, the different types of anemia were usually not included or addressed by these studies. Therefore, it is unclear whether the type of anemia might affect this association.

Despite clear evidence that preoperative anemia is associated with worse outcomes after cardiac surgery, the underlying pathomechanism remains to be determined. Possible explanations include increased requirements of allogeneic blood transfusion, reduced oxygen transport capability, and the consequences of underlying comorbidities, all of which might affect patient outcome as single factors or in combination [5,11]. While RBC transfusion is generally accepted as risk factor for increased postoperative mortality, the evidence of worse outcome due to RBC transfusion in patients with preoperative anemia undergoing cardiac surgery is less clear [15,48]. LaPar and colleagues demonstrated a strong association of RBC transfusion and increased postoperative adverse outcome independent of the preoperative hemoglobin level [43]. In contrast, newer studies could not confirm such an additional negative effect of RBC transfusion in anemic patients [37,49]. Finally, two large RCTs randomizing patients to liberal and restrictive RBC transfusion regimen found no difference in morbidity and mortality with restrictive or liberal transfusion [50,51]. Given these controversial findings, it seems prudent to restrictively administer RBC to patients with hemoglobin <75 g/L during surgery and in the postoperative setting as long as the patient is not bleeding, is cardiovascularly stable, and shows no signs of reduced tissue oxygenation. In patients with acute cardiac ischemia, however, the higher hemoglobin threshold of 90–100 g/L might have benefits [52], potentially due to improved oxygen delivery.

Finally, relevant comorbidities are commonly more prevalent in anemic than in nonanemic patients [5,37]. Anemia definitively appears to be a marker of poorer baseline health status or severe patient illness before cardiac surgery. Accordingly, anemic patients tend to receive not only more RBCs but also more platelet components and other hemostatic products [9,53]. The increased mortality in anemic patients after cardiac surgery might be explained by the underlying comorbidities [15]. Such factors can often not be adequately evaluated in cohort studies. In contrast to this assumption, the meta-analysis conducted by Lau and colleagues found that surgical factors and patient comorbidities including diabetes, age, chronic obstructive pulmonary disease, body mass index and reduced heart function did not significantly affect the risk of morality in anemic patients [39]. This is surprising, as such comorbidities and risk factors are important contributors in most surgical risk calculators such as the EuroSCORE [54].

Finally, sex disparities must be kept in mind when dealing with the impact of anemia on postoperative complications. In fact, Lau and colleagues found a lower mortality in studies with a larger proportion of male patients [39]. Female sex is a risk factor for worse outcome after cardiac surgery, although the pathomechanism is not fully clear [18]. Some authors have attributed it to the lower total RBC mass in female patients and more frequent anemia, finally leading to a higher risk of perioperative blood transfusion [18,22,39].

### 4.2. Non-Anemic Iron Deficiency and Outcome

Iron deficiency can be found in about one to two thirds of preoperative patients scheduled for cardiac surgery [7,55]. The impact of preoperative non-anemic iron deficiency on postoperative adverse outcome under discussion [8,25]. It is biologically reasonable that iron deficiency might have similar impact as anemia, because iron plays an important role in erythropoiesis and in the cellular respiratory chain. Further, the risk of becoming anemic during the preoperative waiting period is increased in patients with non-anemic iron deficiency [7,8]. Accordingly, patients with preoperative iron deficiency are likely to be at a higher risk for perioperative RBC transfusion [7,8].

A retrospective study reported prolonged hospital LOS and fewer days alive at home in patients with non-anemic iron deficiency undergoing cardiac surgery [56]. In agreement, a large prospective cohort study reported that preoperative abnormal iron status defined as absolute iron deficiency, functional iron deficiency, or iron sequestration was associated with increased risk of postoperative major complications [57]. In contrast, other prospective cohort studies found no association of preoperative non-anemic iron deficiency and postoperative complications, LOS, and mortality [7,25]. Further, a recent meta-analysis including 8 studies with a total of 2683 patients with non-anemic iron deficiency before cardiac surgery reported an association with increased requirements for allogeneic RBC transfusion in the perioperative period. However, non-anemic iron deficiency had no impact on mortality, postoperative complications, ICU and hospital LOS, and hospital readmission [7]. The controversial findings might be explained by differences in included patient populations, varying comorbidities, and different types of iron deficiencies (absolute vs. functional) [57]. Accordingly, the reported higher risk of major postoperative complications such as AKI or sepsis but not of other new onset clinically significant disabilities might be explained by other factors than iron deficiency alone [7,8].

Based on the missing evidence for relevant improvement in outcomes, a recent experts’ recommendation stating that all patients undergoing cardiac surgery should be evaluated for iron deficiency, and that iron should be substituted in all cardiac surgery patients is at least questionable [55].

## 5. Intervention in Anemic and Iron-Deficient Patients Before Cardiac Surgery

To allow a targeted anemia therapy and potentially reduced perioperative RBC transfusions and improved patient outcome, the proper and timely diagnosis of anemia is essential [55]. Iron deficiency is common, and iron is essential for the formation of hemoglobin allowing binding and transport of oxygen to end organs. Accordingly, preoperative iron replenishment seems a primary goal in preoperative optimization and is a IIA recommendation in a recent PBM guideline [13]. It can be achieved by dietary changes, oral replacement, or administration of intravenous iron. Each means of iron supplementation has its own onset time, logistic and institutional efforts, and risks [13].

The commonly used oral iron salts have a low bioavailability [58]. To reach a significant increase in hemoglobin levels of ≥20 g/L, an estimated absorption of 500 mg iron is necessary [59]. This amount can be achieved with daily oral intake for at least 4 weeks. Finally, the recent PBM guideline suggests postponing cardiac surgery to allow for oral iron supplementation over 1 to 3 months [13].

Modern oral iron formulations including ferric maltol or sucrosomial iron are preferred. They are widely available, inexpensive, well tolerated and highly effective [58,60]. Usually, a daily dose of iron is prescribed, but administration of oral iron on alternate days might result in a similar or even enhanced absorption [61,62]. As compared to traditional iron salt formulation, sucrosomial iron might have the advantage of excellent tolerability [63], less side effects, and a mostly hepcidin-independent absorption. Therefore, sucrosomial iron might result in better efficacy than ferrous salts in patients with anemia of chronic disease. It has been suggested that sucrosomial iron is an effective treatment of iron deficiency in different clinical settings including PBM and might be as effective as intravenous iron in the perioperative setting [58]. However, sucrosomial iron is usually considered a dietary supplementation rather than a medical drug. Accordingly, costs are generally paid by the patients themselves, which might relevantly affect patient compliance.

**Figure 3:**
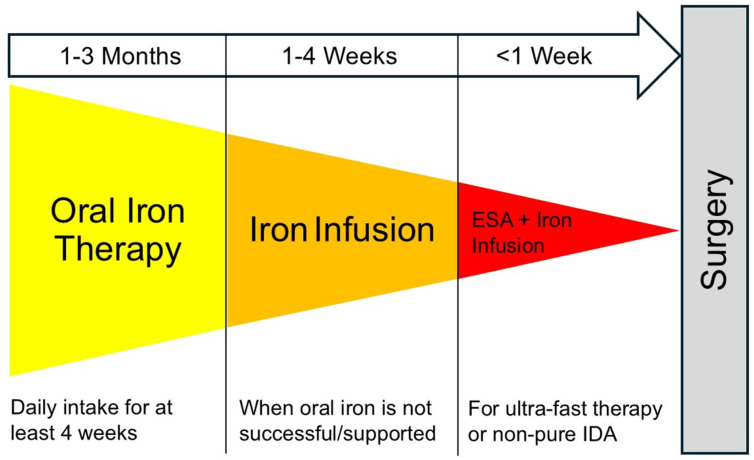
Three steps for perioperative PBM Interventions by anesthesiologists and cardiac surgeons. Suggested escalating schema for perioperative iron therapy and additional therapeutic options. In case of specific hematologic disorders (including inherited hemoglobinopathies), consultation of expert hematologists is advised. Abbreviation: IDA, iron deficiency anemia.

When surgery is scheduled within 2 weeks, oral supplementation is often unsuccessful. In such cases or when gastroenteric absorption might be impaired, intravenous iron administration has been suggested. Finally, functional iron deficiency is more difficult to treat than pure iron-deficiency anemia. It has been suggested to primarily treat the underlying disease, if possible. For an effective increase in the RBC mass, the combination of intravenous iron and erythropoiesis-stimulation agents (ESA) is commonly advocated in patients with anemia of chronic disease or non-pure iron deficiency [31] (Figure 3).

### 5.1. Evidence for Oral Iron Substitution

A recent large trial randomized 1000 patients scheduled for elective cardiac surgery to receive 60 mg/d sucrosomial iron or placebo [63]. The study showed that patients preoperatively treated with sucrosomial iron had significantly higher preoperative hemoglobin values (about 7 g/L higher than the control group) resulting in a lower rate of blood transfusion (35% vs. 65%). The average number of transfused RBC units was reduced from about 2 to 1 [63]. The study also included non-anemic patients, but iron supplementation had the greatest effect in anemic patients [64]. Despite the fact that evidence is limited to this RCT alone, oral iron supplementation in anemic patients received an IIA recommendation in the most recent PBM guideline from the European Association of Cardio-Thoracic Surgery/European Association of Cardiothoracic Anaesthesiology and Intensive Care (EACTS/EACTAIC) [13].

Finally, ferric maltose has successfully used for the long-term management in patients with chronic iron deficiency, including patients with intestinal bowel disease and chronic kidney disease [60]. Data on its perioperative use, however, are limited.

### 5.2. Evidence for Administration of Intravenous Iron

Intravenous iron administration might be advantageous when time to replenish iron stores by oral iron formulations is limited. Compared to oral iron supplementation, the safety of intravenous iron is comparable, but efficacy is improved [55]. In a recent meta-analysis, infusion of intravenous iron was associated with a relatively small but statistically significant reduction of 0.57 units of transfused RBCs as compared to oral or no iron administration in cardiac surgery patients [65]. However, intravenous iron supplementation is associated with significantly higher costs than oral iron administration. Nonetheless, the recent expert recommendation suggested only the use of intravenous iron for preoperative iron supplementation [55]. Ideally, intravenous iron should be given at least two weeks before surgery. However, it might still be effective when given a few days to one day before surgery [66].

The above-mentioned recent meta-analysis including 14 RCTs with a total of 2043 patients found a significant reduction in perioperative RBC transfusion (relative risk (RR) 0.77, 95% CI 0.65–0.91, *p* = 0.002) by preoperative intravenous iron supplementation in cardiac surgery [65]. In addition, postoperative hemoglobin levels were higher in the iron group (mean difference 1.7 g/L, 95% CI 0.6–2.9 g/L). However, there was no significant difference in relevant outcomes including mortality, postoperative complications, and hospital LOS [65]. In contrast, a former meta-analysis in anemic patients with intravenous iron supplementation before cardiac surgery including 6 RCTs (936 patients) and 5 observational studies (1350 patients) found no reduction in transfusion requirements but a decreased mortality (RR 0.58, 95% CI 0.36–0.95, *p* = 0.03) [28]. This mortality benefit, however, disappeared when RCTs alone were included.

### 5.3. Evidence of Combined Therapy with ESA

Two recent RCT showed the efficacy of a combined intravenous iron and ESA therapy as compared to oral or no iron in patients undergoing cardiac surgery [66,67]. In the first study, 505 patients with either preoperative anemia or iron deficiency were randomized to receive either 20 mg/kg ferric carboxymaltose, 40,000 U subcutaneous erythropoietin alfa, 1 mg subcutaneous B12, and 5 mg oral folic acid or placebo on the day before surgery [66]. The study found a significant reduction by one RBC unit in the intervention group, as well as higher postoperative hemoglobin values and reticulocyte counts. No difference in adverse events or mortality was found [66]. In the second study, 156 patients with iron deficiency anemia before elective cardiac surgery were randomized to intravenous ferric derisomaltose 1000 mg and darbepoetin 200 μg subcutaneously or oral ferrous sulphate 600 mg daily for several days [67]. The intervention groups showed significantly higher hemoglobin values, lower risk of transfusion (adjusted RR 0.77; 95% CI 0.63–0.94; *p* = 0.010), but no difference in pre-specified important clinical outcomes and adverse events [67]. A larger retrospective study from Canada evaluated the optimal dosing of ESA and intravenous iron before cardiac surgery found that the preoperative intravenous iron >600 mg and epoetin alfa >80,000 were each associated with significant increases in preoperative hemoglobin values and, hence, lower likelihood of RBC transfusion [68].

Finally, two recent meta-analyses evaluated the perioperative administration of ESA without additional iron administration [69,70]. The first meta-analysis included eight RCTs with a total of 610 patients [69]. In five of these studies, ESAs were administered after anesthesia induction but before surgical incision. In the other three studies with preoperative administration, ESAs were associated with lower intraoperative RBC transfusion (mean difference −0.3; 95%CI −0.55–0.05) [69]. No effect on mortality was found. A more recent meta-analysis included 14 RCTs with a total of 2294 patients found a low certainty that ESA might reduce the need for RBC transfusion with a number needed to treat of 5.6 (95%CI 3.9–12.5) [70]. However, ESA administration might increase the risk of perioperative myocardial infarction [70]. In summary, ESA administration in combination with iron might be associated with lower RBC requirements. However, pure ESA administration might be indicated in selected patients with chronic kidney disease [15,69].

### 5.4. Evidence for Iron Supplementation in Non-Anemic Iron Deficiency

The evidence for iron supplementation in non-anemic iron-deficient patients is scarce. This might be explained, at least in part, by the limited impact of non-anemic iron deficiency on relevant outcomes [25]. However, iron-deficient patients are at higher risk for developing anemia before and after cardiac surgery, and, therefore, are at increased risk for RBC transfusion [8,56]. In fact, a RCT including 200 non-anemic patients scheduled for cardiac surgery reported that a single preoperative intravenous administration of 1 g of ferric carboxymaltose significantly reduced perioperative RBC transfusions (mean ± SD 0.4 ± 0.8 vs. 1.6 ± 4.4; *p*= 0.007) and improved hemoglobin recovery 6 weeks after surgery [71]. Interestingly, the included patients were not necessarily iron-deficient. In agreement with this RCT [71], a recent meta-analysis in non-anemic patients with iron deficiency undergoing major surgery found reduced requirements for RBC transfusion with preoperative intravenous iron supplementation in major cardiovascular surgery but no benefits in mortality, postoperative morbidity, or hospital LOS [72].

### 5.5. Evidence for Postoperative Iron Supplementation

A recent meta-analysis including 15 RCTs with 1865 patients undergoing major surgery showed that intravenous iron administered within 30 days after surgery with the intention to treat postoperative anemia increased hemoglobin levels effectively, whereas oral iron had no beneficial effect. However, no differences in the secondary outcomes including requirements for RBC transfusion, incidence of adverse events, or mortality was found [73]. Data from cardiac surgery are limited. A recent study suggested that postoperative iron supplementation, either with oral sucrosomial iron or intravenous ferric carboxymaltose, might improve functional capacity in the early postoperative period as evident in the enhanced 6 min walking test [74]. No other relevant outcomes were reported in that study.

### 5.6. Strategies for Non-Elective Cardiac Surgery

Evidence for interventions or triggers before urgent surgery is scarce. However, a recent RCT with combined administration of intravenous iron, ESA, vitamin B12 and folic acid on the day before surgery showed reduced requirements for RBC transfusion and higher postoperative values of hemoglobin, CHr, and reticulocyte count [66]. In non-anemic iron-deficient patients, short-term preoperative intravenous iron administration did not affect RBC transfusion, hemoglobin levels, or postoperative outcomes [75]. Future studies must evaluate whether the preoperative administration of iron or ESA should be considered in urgent cardiac surgery. Further, the preoperative transfusion with RBC in anemic patients is not recommended [13]. Finally, postoperative iron infusion might be considered in patients with preoperative anemia undergoing urgent surgery.

## 6. Cost-Effectiveness of Preoperative Interventions to Treat Anemia

As blood products are valuable and limited resources, PBM interventions seem ethical to save blood products and to avoid side effects of unnecessarily administered blood products [11,76]. The cost-effectiveness of PBM interventions, however, has been questioned [77]. The investments might not be fully re-compensated by cost savings due to fewer RBC transfusions, fewer adverse outcomes, and shorter hospitals LOS [11]. Of note, most RCTs and newer retrospective analyses on preoperative PBM interventions show no relevant benefits except reduced blood transfusions. Nonetheless, several recent studies have reported positive cost–benefit effects for preoperative oral and intravenous iron substitution in patients with iron deficiency anemia undergoing general major surgery including cardiac surgery [63,78,79]. However, additional administration of ESA, which is generally necessary in more complex anemias, might mitigate positive cost–benefit effects. Further, it should be noted that preoperative iron supplementation or administration of ESA are not covered by many public health systems, and its cost must be fully borne by the patient [15,80]. In summary, evidence for cost-effectiveness is available for timely supplementation with oral iron and potentially also for intravenous iron administration [11,81].

## 7. Conclusions and Future Direction

Given the relevant consequences of preoperative anemia on outcome in patients scheduled for cardiac surgery, it seems evident that anemia should be recognized as a serious and potentially treatable medical condition instead of a simple laboratory abnormality. Ignoring preoperative anemia should no longer be an accepted clinical practice [11,12,35,55]. Individualized therapy based on diagnostic testing and targeted interventions should be applied rather than general iron supplementation or ESA administration [36].

Future studies need to correctly identify those patients who will benefit most, the optimal treatment strategy including iron in combination with other agents, and the optimum time frames for interventions [82]. In the meantime, it seems prudent to timely treat preoperative iron deficiency anemia in patients undergoing cardiac surgery with modern oral iron formulation such as sucrosomial iron as first-line intervention. In case of patient intolerance or time restriction, intravenous iron administration is recommended. Finally, the combined therapy of intravenous iron and ESA might be applied (Figure 3). The latter has been shown to be safe and most effective to reduce RBC transfusion. However, questionable benefits on morbidity and mortality and limited cost-effectiveness might be the major drawbacks.

## Figures and Tables

**Table 1:** Common types and etiologies of anemia.

Type	Etiology
Iron deficiency anemia	Excessive bleeding - Menstrual Bleeding - Gastrointestinal bleeding (NSAID, aspirin, gastrointestinal pathologies including cancer, etc.)
	Reduced intake - Diet deficient in iron - Poor absorption of iron (intestinal diseases, previous gastrointestinal surgery)
Anemia of chronic disease	Chronic kidney disease Chronic inflammation Rheumatoid diseases Inflammatory bowel disease Congestive heart failure
Megaloblastic anemia	Diet low in vitamin B12 or folate Poor gastrointestinal absorption
Inherited blood disorders	Sickle cell disease Thalassemia

Abbreviation: NSAID, nonsteroidal anti-inflammatory drugs.

**Table 2:** Laboratory characteristics of common types of anemia.

Laboratory Test	Iron-Deficiency Anemia	Non-Anemic Iron Deficiency	Anemia of Chronic Disease
Hemoglobin	↓	normal	↓
Mean corpuscular volume	↓	normal/↓	↓/normal
Reticulocyte count	↓	normal	↓
Ferritin	↓	↓	normal/↑
Transferrin	↑	↑	↓/normal
Transferrin saturation	↓	↓/normal	↓
Soluble transferrin receptor	↑	↑/normal	normal
Reticulocyte hemoglobin content	↓	↓	↓/normal
Erythropoietin levels	↑	↑/normal	normal/slightly ↑
C-reactive protein	normal	normal	↑/normal

Modified after [15,31]. ↓, reduced; ↑, increased.

**Table 3:** Recent large studies * evaluating the impact of preoperative anemia on postoperative outcomes.

Study	Study Design	Sample Size Total/Anemic (%)	Reported Outcome	Anemic vs. Non-Anemic (% or aHR)	***p***
Hazen (2022) [37]	Prospective nation-wide registry; 16 centers; CABG and valves	35,484/6802 (19)	30-day mortality Any RBC transfusion Renal failure Cardiac complications Neurological complications Infections	2.4% vs. 1.0% 49.1% vs. 17.1% 3.5% vs. 1.5% 34.2% vs. 30.4% 1.3% vs. 1.0% 4.5% vs. 3.0%	<0.001 <0.001 <0.001 <0.001 <0.05 <0.001
Kattou (2022) [40]	Retrospective cohort study; 1 center; CABG and valves	1004/251 (25)	30-day mortality Any RBC transfusion AKI MI Stroke/TIA Respiratory failure	5.2% vs. 1.6% 72.5% vs. 30.1% 15.1% vs. 2.6% 16.3% vs. 4.9% 13.1% vs. 6.1% 10.0% vs. 2.9%	0.005 <0.01 <0.001 <0.001 0.001 <0.001
Ripoll (2021) [41]	Retrospective cohort study; 1 center; CABG and valves	4117/1234 (30)	In-hospital mortality Any RBC Transfusion AKI MI or stroke or PE	0.13% vs. 0.03% 80.0% vs. 41.1% 17.7% vs. 7.8% 5.4% vs. 3.4%	0.300 <0.001 <0.001 0.012
Blaudszun (2018) [42]	Retrospective cohort study; 1 center, only women; CABG and valves	1388/333 (24)	In-hospital mortality Any RBC transfusions	2.1% vs. 1.4% 89% vs. 54%	0.552 <0.001
Dai (2018) [32]	Retrospective cohort study; 1 center; CABG and valves	10,589/2715 (26)	In-hospital mortality Any RBC transfusion Renal failure Prolonged ventilation	1.5% vs. 0.4% 66% vs. 26% 5.3% vs. 1.7% 13.3% vs. 6.1%	<0.001 <0.001 <0.001 <0.001
LaPar (2018) [43]	Retrospective quality data analysis; 19 centers; only CABG	33,411/NA	In-hospital mortality Any RBC transfusion Renal failure	1.5% vs. 1.1% (predicted probability) 22.8% vs. 62.1% (predicted probability) 2.3% vs. 1.3% (predicted probability)	NA NA NA
Oprea (2018) [44]	Retrospective cohort study; 1 center; only CABG	6130/1197 (20)	Long-term mortality (median 6.8 years) AKI	aHR 1.29 (95% CI: 1.15–1.44) aHR 1.23 (95% CI: 1.13–1.33)	<0.001 <0.001
Tauriainen (2017) [45]	Retrospective cohort study; 1 center; only CABG	2099/662 (32)	30-day mortality Any RBC transfusion AKI Stroke ICU LOS (mean ± SD)	6.2% vs. 2.3% 89.6% vs. 55.3% 30.6% vs. 12.1% 3.8% vs. 1.6% 2.9 ± 3.5 vs. 1.9 ± 2.0 days	<0.001 <0.001 <0.001 0.001 <0.001
Klein (2016) [4]	National audit; 12 centers; CABG and valves	19,033/5895 (31)	In-hospital mortality Any RBC transfusion ICU LOS (median (IQR))	5.6% vs. 2.3% 63.9% vs. 36.6% 2 (1–4) vs. 2 (0–4)	<0.001 <0.001 <0.001
Padmanabhan (2016) [46]	Retrospective matched case–control study; 1 center; CABG and valves	2340/1170 (50)	In-hospital mortality Any RBC transfusion Renal replacement Surgical site Infections Prolonged airway support	5.6% vs. 3.5% 58% vs. 38% 13% vs. 8% 9% vs. 7% 15% vs. 12%	0.02 <0.01 <0.01 0.05 0.05
von Heymann (2016) [47]	Retrospective cohort study; single center; CABG and valves	4494/1620 (36); of them 1274 with mild and 346 with severe anemia	3-year mortality In-hospital mortality ICU LOS (median (IQR)	51.2% (severe) vs. 30.8% (mild) vs. 15.0% 15.0% (severe) vs. 8.6% (mild) vs. 3.7% 4 (3–7) vs. 6 (4–11) vs. 10.5 (4–11) days	<0.001 <0.001 <0.001

* Published within the last 10 years and including >1000 patients. Abbreviations: aHR, adjusted hazard ratio; AKI, acute kidney injury; CABG, coronary artery bypass grafting; CI, confidence interval; ICU LOS, intensive care unit length of stay, MI, myocardial infarction; NA, not available; PE, pulmonary embolism; RBC, red blood cell; TIA, transient ischemic attack.
